# The use of a curved internal fixation device in adult pelvic Fractures: Short-Term clinical outcomes in high and low energy fractures

**DOI:** 10.1016/j.jcot.2025.103035

**Published:** 2025-05-09

**Authors:** Anthony Sleiman, Christopher Bejcek, Jeffrey Baker, Jeffrey D. Voigt, Kristin Delfino, Matthew Gardner

**Affiliations:** a Southern Illinois University School of Medicine, Department of Orthopedics and Rehabilitation, 801 N. Rutledge St., Springfield, IL, 62702, USA; b Center for Clinical Research, Southern Illinois University School of Medicine, 801 North Rutledge St., Springfield, IL, 62702, USA; c Medical Device Consultants of Ridgewood, LLC, 41 West Prospect St., Waldwick, NJ, 07453, USA; d Springfield Clinic Orthopedics, 800 North1st St, Springfield, IL, 62702, USA

**Keywords:** CurvaFix, Pelvic fractures, Percutaneous fixation

## Abstract

**Background:**

Current methods of percutaneous fixation for pelvic and acetabular fractures are limited due to the curvature of the pelvic anatomy. The goals of fixation are to reduce pain, improve mobility and decrease length of stay. An implant specifically designed for use in the curved osseous fixation pathways of the pelvis may allow for more stable fixation.

**Objective:**

The objective of this review was to examine the results of a new method of fixation (CurvaFix**®**) for pelvic and acetabular fractures.

**Methods:**

A retrospective chart review of CurvaFix was employed and evaluated for implant fixation, complications, hospital length of stay (LOS), and inpatient mobility and then compared to current standards of care from the literature for percutaneous fixation.

**Results:**

A curved intramedullary device was used to treat 31 low energy and 21 high energy fractures over a median 3.4 months. The median LOS was 7 [1 to 27] days for low and 11 [0 to 68] days for high energy fractures. Median time to first inpatient ambulation was 37 h in high energy fractures and 25 h in low energy injuries. Aggregated complications occurred in 21.3 % (10/46) of patients. Peri-implant failure occurred in 2 patients, with no incidences of hardware failure.

**Conclusions:**

This case series demonstrates curved internal fixation is a viable option to consider among other treatment modalities for pelvic ring and acetabular fractures and may allow for early inpatient ambulation and a shorter LOS. Comparative studies are needed to confirm this.

## 1 Introduction

Fractures of the pelvis and acetabulum are complex, severe, and morbid orthopedic injuries.^1^ High energy trauma such as motor vehicle accidents are a major cause of these injury patterns. Increasingly, geriatric populations are also at higher risk of low energy fragility fractures of the pelvis.^2^ Advanced resuscitation protocols and higher quality care have led to improvement in patient survivability,^3^ suggesting orthopedic surgeons will be increasingly challenged with management of these complex fractures. Pelvic ring fractures are estimated to account for 2–8 % of skeletal fractures.^4^, ^5^, ^6^ Over a 17-year period in the United States, the population-adjusted incidence of pelvic ring fractures significantly increased from 27.24 to 34.30 per 100,000 capita.^7^ Surgical intervention in select pelvic ring fractures is preferable to conservative management as it has been shown that treating pelvic fractures with internal fixation is associated with decreased mortality.^7^

A common surgical intervention for pelvic ring injuries involves placing percutaneous cannulated screws through the bony corridors of the pelvis to achieve fixation.^8^ These intracortical spaces in the pelvic anatomy where internal fixation devices can be used have been characterized as osseous fixation pathways (OFP).^9^ The stability achieved from a screw situated within an OFP is largely based on the longitudinal cross-section of the screw within the cancellous bone. A screw must have sufficient length and diameter to withhold patient forces, so they do not exceed the cancellous bone's yield point.^10^ A straight screw placed in the curved OFP of the pelvis significantly limits the longitudinal cross-sectional area the screw can occupy, thus limiting the stability of the construct. Successful screw fixation is also highly dependent on bone quality.^11^ A curved implant that more closely approximates the curvature of the bony pelvic corridors would allow the implant to be wider and longer, which would in turn improve stability.^12^^,^^13^ The constriction points and radii of curvature of pelvic OFPs can help guide surgeons using curved internal fixation devices.^12^

CurvaFix is a novel FDA cleared intramedullary implant that utilizes four internal cables to allow for flexible insertion into a curved OFP and is indicated for fixation of fractures of the pelvis.^14^

Once in place, the cables are locked to make the construct rigid, thereby providing stability to the fracture. Fig. 2 shows a positioning example of the CurvaFix implant within the pelvis.

**Fig. 1:**
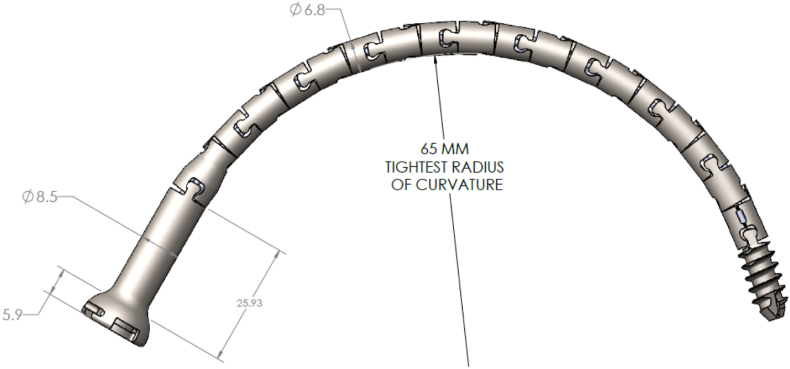
Shows the CurvaFix implant and its radius of curvature for a 180 mm implant.

**Fig. 2:**
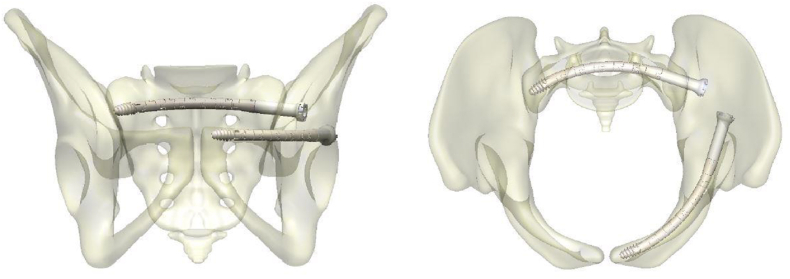
CurvaFix positioning example.

**Fig. 3:**
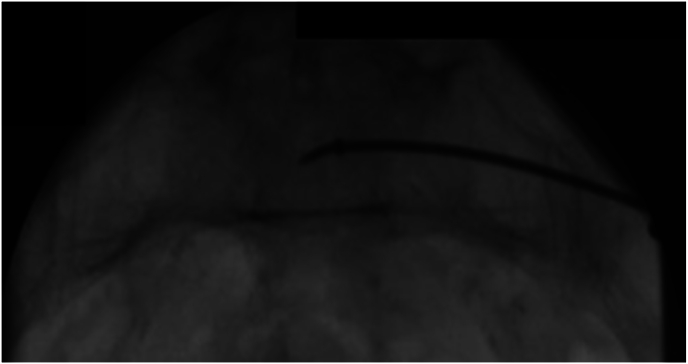
Positioning of the guidewire.

**Fig. 4:**
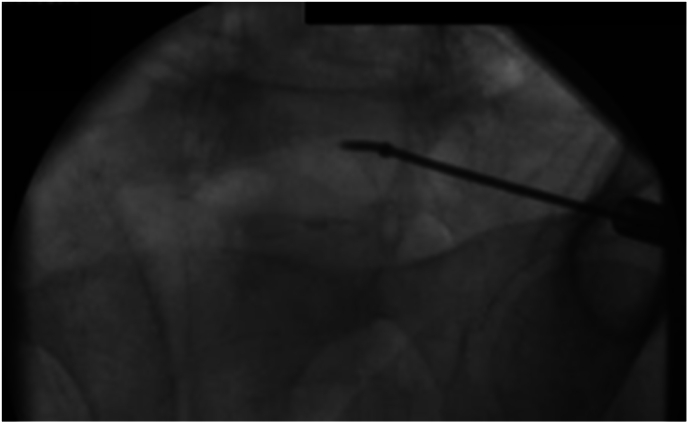
Tracking of the CurvaFix implant along the guidewire.

**Fig. 5:**
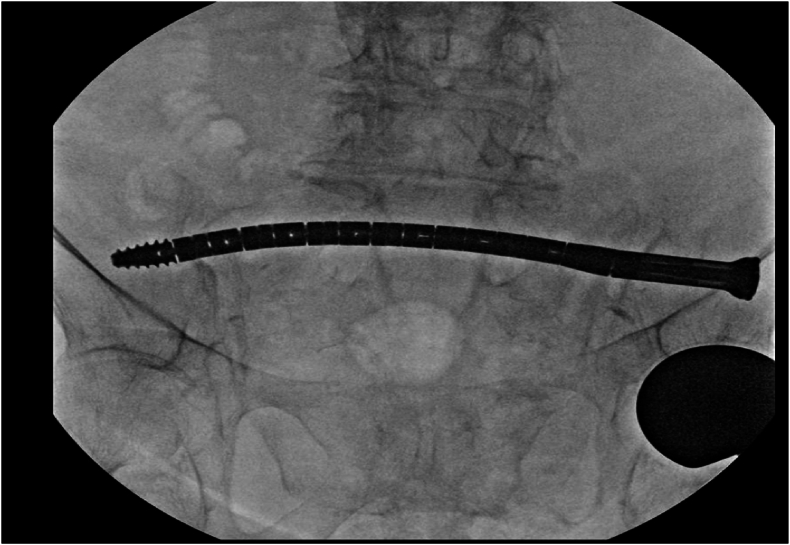
CurvaFix positioned across iliosacral fracture.

**Fig. 6:**
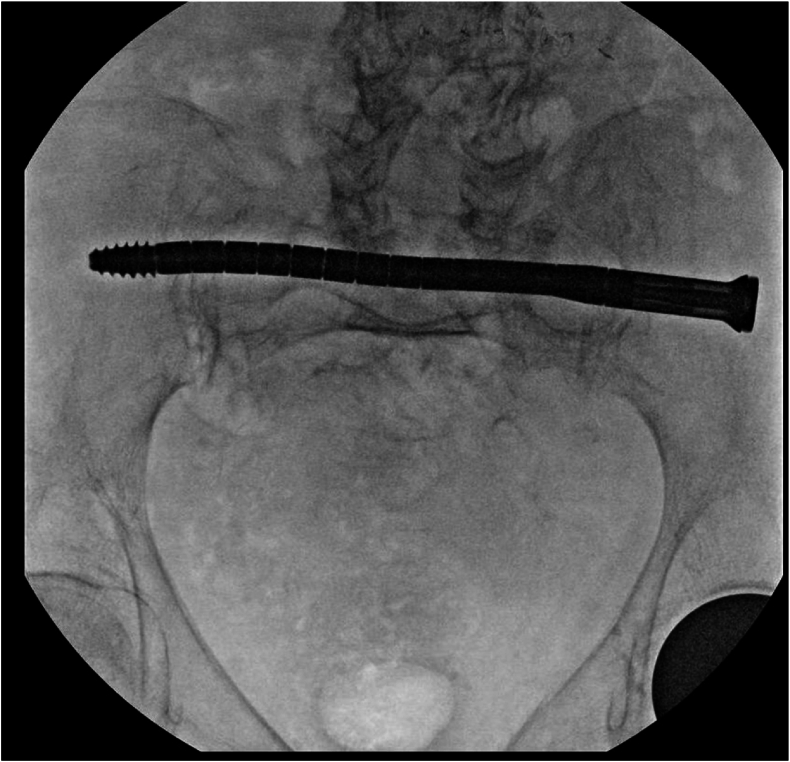
CurvaFix locked in place across fracture.

Fig. 3, Fig. 4, Fig. 5, Fig. 6 provide radiographic intraoperative positioning of the CurvaFix implant during fixation of a iliac/sacral fracture.

The objectives of this study are to assess short-term clinical outcomes in patients who received internal fixation with a curved implant for their pelvic fracture (including failure of fixation, reoperation rates, infection, deep vein thrombosis, pulmonary embolism, and postoperative inpatient ambulation). While there are currently studies supporting the concept of curved internal fixation devices guided by OFPs in the pelvis,^12^ there are no studies describing clinical outcomes and surgeon experience with this specific implant in more than a few patients.^13^

## 2 Methods

A retrospective chart review of patients who suffered pelvic ring injury or acetabular fracture and underwent pelvic ring fixation with a curved intramedullary fixation device between 01/01/2021 and 10/31/2023 was performed. The inclusion criteria used in the analysis were patients aged 18 years or older who sustained a pelvic ring injury or minimally displaced acetabular fracture and underwent primary fixation for their injuries with a curved intramedullary implant. Patients were excluded if fixation was for revision surgery. Institutional Review Board Approval was obtained. All patients who received fixation for Lateral Compression type 1 (LC1) or LC2 pelvic ring injuries previously failed a 2–3 day weight bearing trial with physical therapy prior to undergoing operative fixation. Data was obtained from time of procedure through all available post-operative follow-ups. Electronic medical records were reviewed for baseline demographic information including age at the time of procedure, sex, BMI, and race/ethnicity. Operative notes were reviewed to document the type of fixation, duration of surgery, blood loss, and any intra-operative complications. Operative indications, radiographic information (both initial and during post-op follow up), and all available post-operative office visits were reviewed to assess complications (loss of fixation, nonunion, implant loosening/pullout, infection, deep vein thrombosis, pulmonary embolism, hardware removal, reoperation, ambulation, mortality). Postoperative inpatient ambulation was defined as the ability to ambulate at least 10-feet with 2-wheeled walker and assistance of a physical therapist.

Descriptive statistics were computed for all study variables. Continuous variables were described with measures of central tendency (mean, median) and dispersion (range, standard deviation). Categorical variables were summarized as frequencies and percentages. Chi-square or Fisher's Exact test was used to compare categorical variables. Continuous variables were compared with the student's t-test or Mann Whitney *U* test, depending on distribution. For the longitudinal data, survival curves are estimated using Kaplan-Meier methodology and analyzed with a log rank test to assess return to baseline ambulatory status. P-values ≤0.05 were considered statistically significant. All statistical analysis was performed via SAS v9.4.

## 3 Theory

A curved implant should fit better and provide better fixation of fractured bones of the pelvis and acetabulum. Prior studies have examined the OFPs of the pelvis and acetabulum.^11^^,^^12^ The CurvaFix Implant was designed to fit within these pathways and to lock in place upon confirmation placement. It is the intention of this analysis to report on a much larger series of pelvic and acetabular fracture fixation using percutaneous implantation.

## 4 Results

A total of 55 patients with pelvic and acetabular fractures who received percutaneous internal fixation met initial inclusion criteria. Three patients were excluded due to an implant for revision fixation of their pelvic injuries. The remaining 52 patients were included in the overall analysis. Forty-six patients had follow-up data available for longitudinal analysis. (six patients lost to follow-up) Patients were 68.9 years (range 22–101 years), female (61.5 %), with a mean Charlson comorbidity index^15^ of 4.2. By mechanism of injury, 31 patients (59.6 %) had low-energy (e.g. ground level fall) pelvic injuries and 21 (40.4 %) had a high-energy mechanism (e.g. motor vehicle crash). Most treated pelvic ring injury patterns were lateral compression (LC1) at 36.5 %, and LC2 at 28.8 %. The most frequently treated acetabular fracture was anterior column (7.7 %). The most frequently treated sacral injury was isolated bilateral sacral ala fracture (7.7 %). Seven patients were treated for combination pelvic ring and acetabular fractures; (APC1 and anterior column, LC1 and anterior wall, LC1 and posterior wall, LC2 and associated both column, LC2 and transverse, LC2 and t-type, LC3 and vertical shear). Three patients received fixation for isolated anterior column acetabular fractures. Four patients received fixation for isolated bilateral sacral ala fractures.

When divided by high-energy and low-energy mechanism, low-energy patients were significantly older (79.9 years vs. 52.6 years, p < 0.0001), predominantly female (74 % vs. 43 %, P = 0.0227), exhibited a higher Charlson comorbidity index (CCI 5.7 vs. 2.1, p < 0.0001), had shorter operative time (0.88 h vs. 1.91 h, p = 0.0001), and shorter length of hospital stay (8.5 vs. 14.8 days, p = 0.0474). Baseline demographic information fracture classification, and operative data are summarized in Table 1, Table 2, Table 3.

**Table 1:** Baseline demographics and fracture classification.

**Age at time of surgery, Mean (SD)**		68.85 (20.0)
**BMI, Mean (SD)**		25.83 (10.7)
**Charlson Comorbidity Index (CCI), Mean (SD)**		4.23 (2.9)
**Sex, n (%)**	Male	20 (38.5)
	Female	32 (61.5)
**Comorbidities, n (%)**	COPD	11 (21.2)
	Myocardial Infarction	7 (13.5)
	CHF	8 (15.4)
	PVD	16 (30.8)
	Diabetes	15 (28.8)
	CVA/TIA	6 (11.5)
	CKD	3 (5.8)
	Connective Tissue Disorder	0
	Liver disease	2 (3.8)
	Peptic Ulcer Disease	1 (1.9)
	Hemiplegia	0
	Dementia	6 (11.5)
	Solid Tumor, Leukemia, Lymphoma	2 (3.8)
	AIDS	0
**Smoking status, n (%)**	Current	11 (21.2)
	Former	11 (21.2)
	Never	28 (53.8)
**Mechanism of Injury, n (%)**	High Energy (MVC)	21 (40.4)
	Low Energy (GLF)	31 (59.6)
**Young & Burgess Classification, n (%)**	LC-1	19 (36.5)
	LC-2	15 (28.8)
	LC-3	1 (1.9)
	APC-1	2 (3.8)
	APC-2	2 (3.8)
	Vertical Shear	1 (1.9)
	Combined Mechanism	1 (1.9)
**Acetabular Fracture, n (%)**	Anterior column	4 (7.7)
	Posterior column	1 (1.9)
	Anterior wall	1 (1.9)
	Posterior wall	1 (1.9)
	Associated both column	1 (1.9)
	Transverse	1 (1.9)
	T-type	2 (3.8)
**Sacral Fracture, n (%)**	Isolated bilateral sacral ala fractures	4 (7.7)
	Denis 2	1 (1.9)
	Pathologic fracture	1 (1.9)

BMI = body mass index, SD = standard deviation, COPD = chronic obstructive pulmonary disease, CHF = congestive heart failure, PVD = peripheral vascular disease, CVA = cerebrovascular accident, TIA = transient ischemic attack, CKD = chronic kidney disease, MVC = motor vehicle crash, GLF = ground level fall.

**Table 2:** Baseline demographics by mechanism of pelvic injury.

Sex	High Energy	Low Energy	Total	P-Value
Male	12	8	20	0.0227
Female	9	23	32	
**Smoking Status**				
Never (N)	12	16	28	0.6418 (N vs C + F)
Current (C)	8	3	11	
Former (F)	0	11	11	
**PVD**				
No	18	18	36	0.0340
Yes	3	13	16	
**MI**				
No	20	25	45	0.2194∗
Yes	1	6	7	
**CHF**				
No	20	24	44	0.1225∗
Yes	1	7	8	
**CVA/TIA**				
No	20	26	46	0.3818∗
Yes	1	5	6	
**COPD**				
No	19	22	41	0.1651∗
Yes	2	9	11	
**Diabetes**				
No	18	19	37	0.0565
Yes	3	12	15	
**Liver Disease**				
No	20	30	50	0.9999∗
Yes	1	1	2	
**CKD**				
No	20	29	49	0.9999∗
Yes	1	2	3	
**PUD**				
No	20	31	51	0.4038∗
Yes	1	0	1	
**Dementia**				
No	20	26	46	0.3818∗
Yes	1	5	6	

PVD = peripheral vascular disease, MI – myocardial infarction, CHF = congestive heart failure, CVA = cerebrovascular accident, TIA = transient ischemic attack, COPD = chronic obstructive pulmonary disease, CKD = chronic kidney disease, PUD = peptic ulcer disease.

∗Denotes Fisher's Exact Test.

**Table 3:** Demographics and operative data by mechanism of pelvic injury.

Measure	Mechanism	N	Mean	Std Dev	Median	Minimum	Maximum	P-Value
BMI	High Energy	21	26.2	6.8	25.1	19.2	43	0.6831
	Low Energy	28	25.6	4.0	26.3	17.2	34.1	
Age	High Energy	21	52.6	19.5	57	22	84	<0.0001
	Low Energy	31	79.9	10.8	81	51	101	
Charlson Comorbidity Index	High Energy	21	2.1	2.6	1	0	9	<0.0001
	Low Energy	31	5.6	2.2	5	1	11	
Surgery Duration (Minutes)	High Energy	21	114.4	70.2	87	28	245	0.0001∗
	Low Energy	31	52.6	28.0	50	24	169	
Blood Loss Amount (cubic centimeters; cc)	High Energy	19	201.1	146.7	150	20	500	0.0766∗
	Low Energy	30	130	96.1	100	25	400	
LOS (Days)	High Energy	21	14.8	15.0	11	0	68	0.0474∗
	Low Energy	31	8.5	5.3	7	1	27	

∗Denotes Mann Whitney *U* test.

All patients received operative intervention with percutaneous fixation of their fractures. The median procedure duration was 52.5 min [24–245]) with median blood loss of 100 cc [20 cc–500 cc]. Patients remained hospitalized for a median of 8.0 days [0-68].

At pre-injury baseline,^e^ 37 patients (71.2 %) were independent ambulators, 10 patients (19.2 %) used a 2-wheeled walker, 3 patients (5.8 %) ambulated with a cane, and 2 patients were wheelchair bound (3.8 %). Postoperatively, most patients (61.5 %) were ambulating with a walker at the time of hospital discharge.

Forty-eight patients had available inpatient timed ambulation data. Patients ambulated at least 10 feet with a two-wheeled walker and the assistance of physical therapy in a median of 26.7 h following surgery [7.6–458.7 h], with low energy patients achieving this sooner than high energy, although not statistically significant p = 0.0539 (Table 4).

**Table 4:** Time in **hours** to first inpatient ambulation^a^.

Cohort	N	Mean	Median	Std Dev	Q1	Q3	IQR	Min	Max
All	48	57.8	26.7	77.1	23.4	49.7	26.4	7.6	458.7
High	18	88.3	37	114.1	25.3	100.8	75.5	15.6	458.7
Low	30	39.5	25.2	32.8	22.7	44.3	21.5	7.6	144.8

a Ambulation 10-feet with 2-wheeled walker and assistance of a physical therapist.

Of the 46 patients with follow up, median follow up time was 3.4 months [0.4–11.8] (high energy: 4.6 months [0.4–9.9] and low energy 2.8 months [0.6–11.8]).

**Fig. 7:**
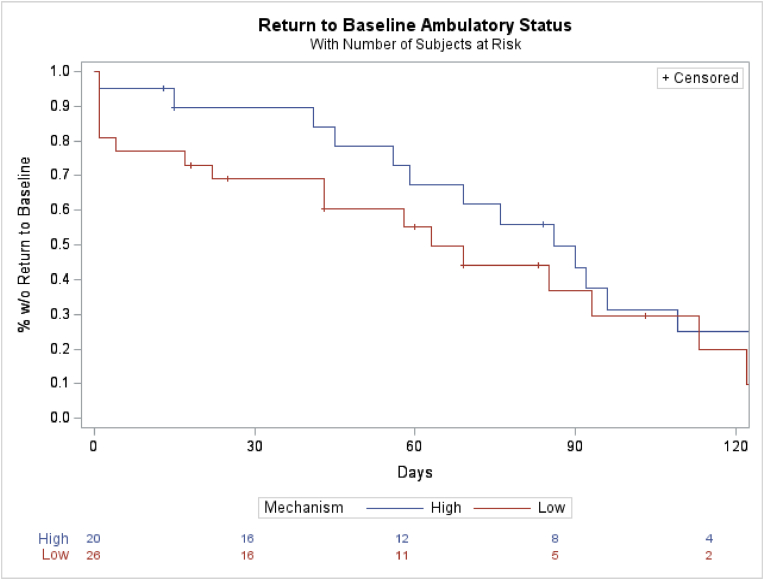
Percent of patients who did not return to baseline ambulatory status over 4 months.

At 4 months,the probability of not having returned to baseline was 24.8 % ± 10.6 % in the high energy and 20.7 % ± 10.6 % in the low energy cohort (Fig. 1). A total of 32 patients (70 %) with high and low energy pelvic fractures achieved baseline ambulatory status in a median of 58.5 days [1–138 days].

(Table 5). The median follow up time for the 14 patients (30 %) who did not achieve baseline ambulatory status was 64.5 days [13–252 days]. There was no significant difference between high and low energy mechanism groups (log rank p-value = 0.3986). The percent of patients who did not return to baseline ambulatory status over 4 months is found in Fig. 7 below.

**Table 5:** Time from surgery to return to baseline ambulatory status (days).

Cohort	N	Mean	Std Dev	Median	Min	Max
High	15	73.7	38.9	76	1	138
Low	17	43.4	42.0	43	1	122

### 4.1 Complications

Of the n = 46 patients with follow up information, a total of n = 10 (21.3 %) experienced 1 or more post-operative complications. The breakout of the number of complications by patient can be found in Table 6.

**Table 6:** Complications.

Patient (# complications)	Complications
A (1)	Major bleeding requiring transfusion
B (1)	Major bleeding requiring transfusion
C (1)	Major bleeding requiring transfusion
D (2)	Major bleeding requiring transfusion; Deep Vein Thrombosis (DVT)
E (3)	Major bleeding requiring transfusion; Repeat surgical intervention; Hardware removal
F (2)	DVT; Pulmonary Embolism (PE)
G (2)	Major bleeding requiring open or endovascular intervention; Major bleeding requiring transfusion
H (1)	PE
I (2)	Peri-implant failure; Repeat surgical intervention
J(1)	Peri-implant failure

There were no instances of post-operative infection, antibiotics administered, nonunion, osteonecrosis, malunion, or hardware failure observed in the retrospective analysis.

There was one incidence of bleeding requiring endovascular embolization, six incidences of bleeding requiring transfusion, two incidences of deep vein thrombosis (DVT), and two incidences of pulmonary embolism (PE). All thromboembolic complications were treated with oral factor X inhibitors and subsequently resolved. There were two cases of peri-implant failure and one reoperation (described in Appendix A).

## 5 Discussion

There is a sparsity of high-quality literature and a lack of consensus regarding treatment of low energy pelvic ring injuries, especially given the wide spectrum of injury. Furthermore, there are differences in the definitions of instability. Literature has supported operative fixation to improve pain, mobilization, and length of stay. Some literature has found low rates of further displacement with nonoperative treatment of minimally displaced sacral fractures and pelvic ring injuries, with no differences in mobilization. However, much of the existing prospective, comparative literature has shown improvement in pain (low and high energy),^7^^,^^16^^,^^17^ morbidity (high energy),^18^ mobilization.

(high energy)^16^ and improved 1 and 2-year survival rate with operative fixation(low energy).^7^^,^^19^, ^20^, ^21^, ^22^ Percutaneous fixation has been associated with fewer intraoperative complications (less surgical trauma, less blood loss, lower infection rates) and shorter length of hospital stay compared to open reduction internal fixation of some pelvic ring injuries, including Day type II crescent fracture dislocations.^23^^,^^24^ In open anterior and posterior surgically treated patients with pelvic ring injuries overall complication rates can be upwards of 34–44 % per patient.^25^ In this case series there was a 21.3 % complication rate.

Peri-implant failure of the curved intramedullary device was 3.8 % (2/52). One study on the use of screw fixation in pelvic ring and sacral injury have demonstrated a 4.5 % failure rate with similar follow-up.^26^

Hospital length of stay (LOS) in high energy and low energy fractures was 14.76 ± 15 days and 8.45 ± 5.32 days, respectively. Other studies examining LOS in similar low energy fractures show LOS of 10.6 ± 9.5 N = 31) days in surgically treated patients with internal fixation.^17^ Other studies examining the LOS in high energy pelvic fractures with co-injuries (internal bleeding and abdominal injury) similar to the CurvaFix cohort had hospital LOS on average of 17–22 days^27^ This requires further investigation with comparative studies in the use of curved intramedullary fixation versus other internal fixation methods.

The short-term clinical outcomes in patients who received percutaneous fixation with a curved intramedullary device demonstrate that operative fixation is safe and allows early inpatient ambulation. Weight bearing in the management of pelvic fractures has low-quality mixed evidence in systematic reviews, and no timeline exists recommending when patients can begin ambulating after either nonoperative or operative management.^28.^ The literature appears to have some consensus on restrictions on the time frame for those who should not weight bear (i.e. Tile Type C fracture^29^ – those that are vertically unstable as well as rotationally unstable) of 8–9 weeks.^26^ However, there is a lack of consensus and evidence on weight bearing regimens and when it may be advisable to do so.^28^^,^^30^ Early weight bearing has a number of benefits -including reductions in bone loss, muscle loss, and joint stiffness, earlier discharge, and reduced venous thromboembolism.^26^ The drive towards early weight bearing in other anatomic areas has led to similar advancements in treatment options for pelvic ring injuries to afford patients these same benefits. The results in this study suggest curved intramedullary implants may be a useful tool to consider versus other treatment modalities to help improve outcomes in patients with pelvic ring and acetabular injuries.

Advantages to screw fixation: Due to the nonlinear structure of the CurvaFix device, it prevents rotation around the device, where if screws were employed, multiple screws would be required to prevent rotation. The intramedullary corridors of the pelvis are curved in nature.^9^ The CurvaFix implant can follow and connect these corridors allowing for longer implants and for increased stability of fracture fixation.

Potential disadvantages: There is a learning curve with the CurvaFix implant which may be longer than with percutaneous screw fixation.

Limitations of analysis: This retrospective heterogeneous case series is limited to short-term outcomes and lacks a comparison group. Follow-up data is also limited to the short post-operative period. However, early clinical outcome data support the viability of curved intramedullary fixation with several possible advantages including a percutaneous approach, extension of fixation into the pubic rami, short operative times, and early mobilization. This study may help lay the groundwork for the development of treatment guidelines for patients with pelvic ring injuries.

## 6 Conclusion

The short-term clinical outcomes in patients with pelvic ring and acetabular injuries who received percutaneous fixation with a curved intramedullary device demonstrate that operative fixation is safe and allows early inpatient mobilization. The results in this case series suggest these implants may be a useful tool to consider among other operative treatment modalities. Further research is needed to compare treatment with other strategies on both clinical and cost effectiveness perspectives.

## Guardian/patient's consent

The study was a retrospective review of patient records who had undergone percutaneous fixation of pelvic and acetabular fractures. IRB approval only was required which was approved by the institution.

## Author contributions

AS, CB, and MG wrote draft of manuscript, JV and AS edited and rewrote manuscript. KD and JV performed statistical analyses. JB assisted with data collection and IRB submission. All authors reviewed the final manuscript and approved it.

## Ethics in publishing

IRB clearance for this retrospective analysis was approved by Southern Illinois University School of Medicine.

## Funding statement

Jeffrey D. Voigt, one of the co-authors, received funding from CurvaFix to help in the analysis and writing of the manuscript.

## Declaration of competing interest

The authors declare the following financial interests/personal relationships which may be considered as potential competing interests:Dr. Matthew Gardner reports writing assistance was provided by Jeffrey D. Voigt of Medical Device Consultants of Ridgewood, LLC. Matthew Gardner reports a relationship with CurvaFix, Inc. that includes: board membership and consulting or advisory. The other authors, Dr. Christopher Bejcek, Dr. Anthony Sleiman, Dr. Jeffrey Baker and Dr. Kristin Delfino, declare that they have no known competing financial interests or personal relationships that could have appeared to influence the work reported in this paper.

## Appendix

### Appendix A: Descriptions of per-implant failure

There were 2 cases of peri-implant failure - one in which anterior and posterior columns sustained fracture. The anterior column was stabilized with CurvaFix. However the posterior column was nondisplaced and deemed stable - therefore fixation of this osseous pathway was withheld. The femoral head collapsed into the medial wall of the acetabulum without displacement of the fixed anterior column and with displacement the unfixed posterior column resulting in acetabular protrusion. The patient was subsequently converted to THA without removal of the anterior column CurvaFix. The other patient received percutaneous intramedullary fixation across right trans-sacral-trans-iliac column, and anterior ring fixation with nitinol staples across the pubic symphysis for their vertical shear pelvic injury. The patient suffered an inpatient ground-level fall, resulting in loss of fixation of the pubic symphysis, and required subsequent reoperation for hardware removal and revision with open reduction and internal fixation (ORIF) of the pubic symphysis with plate and screws. However trans-sacral-trans-iliac fixation was well maintained and did not require revision. There was one other reoperation for hardware removal due to post-operative CT scan demonstrating malposition of the implant. The hardware removal occurred the same day as initial fixation and there were no further sequelae.

## Footnotes

e Pre-injury baseline is their level of ambulation before suffering the pelvic ring injury. It is a question asked to all patients by physical therapy and documented in the EMR in their notes.
